# The AM4I Architecture and Framework for Multimodal Interaction and Its Application to Smart Environments

**DOI:** 10.3390/s19112587

**Published:** 2019-06-06

**Authors:** Nuno Almeida, António Teixeira, Samuel Silva, Maksym Ketsmur

**Affiliations:** 1 Department of Electronics, Telecommunications and Informatics, Universidade de Aveiro, 3810-193 Aveiro, Portugal; nunoalmeida@ua.pt (N.A.); sss@ua.pt (S.S.); mvk@ua.pt (M.K.); 2 Institute of Electronics and Informatics Engineering of Aveiro, Universidade de Aveiro, 3810-193 Aveiro, Portugal

**Keywords:** smart environments, sensors, devices, human-computer interaction, multimodal interaction, multi-device, adaptation

## Abstract

Technologies, such as smart sensors, actuators, and other kinds of devices, are often installed in our environments (e.g., our Homes) and available to integrate our daily lives. Despite their installation being motivated by the pursuit of automation and increased efficiency, making these environments usable, acceptable and enjoyable in a sustainable, energy efficient way is not only a matter of automation. Tackling these goals is a complex task demanding the combination of different perspectives including building and urban Architecture, Ubiquitous Computing and Human-Computer Interaction (HCI) to provide occupants with the means to shape these environments to their needs. Interaction is of paramount relevance in the creation of adequate relations of users with their environments, but it cannot be seen independently from the ubiquitous sensing and computing or the environment’s architecture. In this regard, there are several challenges to HCI, particularly in how to integrate this multidisciplinary effort. Although there are several solutions to address some of these challenges, the complexity and dynamic nature of the smart environments and the diversity of technologies involved still present many challenges, particularly for its development. In general, the development is complex, and it is hard to create a dynamic environment providing versatile and adaptive forms of interaction. To participate in the multidisciplinary effort, the development of interaction must be supported by tools capable of facilitating co-design by multidisciplinary teams. In this article, we address the development of interaction for complex smart environments and propose the AM4I architecture and framework, a novel modular approach to design and develop adaptive multiplatform multilingual multi-device multimodal interactive systems. The potential of the framework is demonstrated by proof-of-concept applications in two different smart environment contexts, non-residential buildings and smart homes.

## 1. Introduction

We live in an age where our environment can easily be populated with a wide diversity of technologies. From small sensors/actuators to large home appliances, all connected to networks, they, can be harnessed to improve some aspects of our daily lives [1,2,3] These connected technologies can be used to create pervasive smart environments, and, while if on the one hand the system can be autonomous, in many occasions, making decisions to better help the users, it should also have a pervasive and simple interface providing the user with the necessary means to take control, at any time. These smart environments can be instantiated in different contexts, from smart homes, smart buildings or smart cities. The wider the context, the more complex it gets, with the increasing number of sensors and devices that needs to be integrated and the variety of contexts for which user interaction needs to be supported.

Sensors and actuators can serve different purposes. They can be used to monitor and control the environment, their primary purpose, but can also be used to improve interaction between user and environment. Devices, such as smartphones, which people always carry around, or dedicated devices installed in relevant locations can be used to directly interact with the system, with other dedicated sensors providing context information (e.g., ambient light intensity) to adapt interaction to the environment and task.

The use of smart home devices is already common, e.g., smart lights, which users can control through a portable device, such as a smartphone or tablet, or a virtual assistant, such as Alexa. However, even though these devices support a variety of features, there are several limitations to the way users interact with them and, very importantly, to how they can be integrated in an interactive ecosystem, instead of working as isolated devices, each controlled by its custom interface.

To enable fully exploiting these new scenarios providing a wide range of interaction capabilities to the user, enhancing how it can deal with its complexity and harness the full potential of the smart environment, at reduced costs of development, is a complex task, entailing, among other aspects: design and development of complex distributed approaches; adaptive solutions, to encompass the dynamic nature of the environment (e.g., number of devices changes over time); integration with the occupant’s devices, e.g., smartphones; and adaptation to the user (e.g., accounting for individual differences and preferences) and a dynamic context.

In this article, we propose a novel modular approach to the design and development of multimodal interactive solutions for smart environments. Our approach provides a distributed, ubiquitous, loosely coupled solution, with low demands of local computational resources and the ability to:

(a) exploit the different ways of communication between the environment and the user through seamless integration of user and building (interactive) devices;
(b) consider additional environment information to contextualize interaction (e.g., comfort related measures);
(c) evolve, through time, to encompass novel technologies and interaction designs;
(d) generically support system instantiations complying with different cultures.

A first instantiation of the proposed approach, enabling the design and development of Adaptive Multiplatform Multidevice Multilingual Multimodal Interaction (the AM4I—“amphorae”—framework) is also presented, providing:

(a) overall support to develop and deploy multimodal interaction;
(b) simple integration of user and environment devices and sensors and their dynamic (i.e., changeable over time) management;
(c) integrated exploration of several ways to communicate—the so-called modalities—in both directions between users and the environment;
(d) consideration of multi-device environments as seamless interaction ecosystems; and
(e) provide off-the-shelf modalities, which can be readily available to developers, avoiding the need to master their supporting technologies and complexity including, for instance, speech interaction and multilanguage support.

The remainder of this document starts by providing an overall panorama of multimodal and multidevice interaction and a brief background on sensors and actuators considering their most relevant characteristics for the envisaged scenarios. After the background, the next section presents the main requirements and based on the requirements the AM4I architecture and framework proposal. The following section, presents two demonstrative applications depicting the features and capabilities of the proposed architecture and framework. Finally, the conclusion presents the main achievements and points some routes for future work.

## 2. Background & Related Work

This section presents an overview of research areas that can potentially serve the Smart Environments scenario in its full complexity of occupant and device diversity, covering multimodal interaction, multi-device applications, and evaluation of pervasive ubiquitous systems. In this context, we identify a set of challenges that have, yet, to be explicitly considered and addressed, from a Human-Computer Interaction standpoint, to contribute to unleash the full potential of Smart Environments. These consubstantiate the contributions presented in this article.

### 2.1. Multimodal Interaction

Such as humans communicate with each other naturally, using multiple senses, communication with machines (and buildings) should become more natural and encompass such diversity of options. Multimodal Interaction (MMI), i.e., interaction considering a wide set of possibilities, such as touch, speech, and gestures, has become a possibility given the currently available technology [4].

The literature describes several MMI architectures and frameworks supporting application development considering different approaches such as agents [5,6], components [7,8], layers [9], and server-based [10,11]. However, these architectures lack the modularity that would enable a truly generic approach since, in many aspects, components are tightly coupled, making them hard to expand, replace, or reuse in different applications. This issue is further emphasized by the diversity of architectures and how their different components communicate. For some modalities, e.g., speech, if a developer wants to enable it for different systems, he needs to deal with the complexity of each system and most frequently implement a custom modality for each. Therefore, only a limited number of modalities will be available for each framework (and application) and the lack of initial support for a basic set of modalities will prevent developers from fully adopting it, given the high entry cost, and is far from enabling the full potential of multimodal applications. The diversity and complexity of the existing technologies supporting the modalities requires the ability of the multimodal systems to deal with new technologies transparently, easily adapting to new modalities, and that developers are able to integrate new modalities without the knowledge of their internal complexity [12].

Here is where the W3C standards for MMI can be a valuable asset [13,14]. These propose a new standard for multimodal architectures defining standard markup for communication and specifying a set of internal components of the architecture. Despite its focus on web scenarios, and some open questions, such as how to accomplish the discovery of new modalities available in the system, it seems as a good starting point to create multimodal applications as it serves the principles of a decoupled architecture, enabling easy integration of modalities, and is open enough to encompass new evolutions. These standards have inspired the proposal of frameworks such as OpenStream Cue-me (http://www.openstream.com/cueme.html) and have also been considered in our work when we extended our agent-based architecture for Ambient Assisted Living (AAL) [15,16,17].

Multiple modalities, widening the range of interaction options, is only a first step in supporting multimodality. The joint use of multiple modalities for input—e.g., pointing to a folder while saying “open this”—is paramount to attain a more natural interaction. Modality fusion has attained maturity [18], but fusion engines are not yet integrated in MMI architectures. Our team recently presented a first proposal regarding how to integrate a fusion engine in a W3C-aligned MMI architecture [19], but to tackle fusion in the generic decoupled scenario of an MMI architecture, where a wide range of modalities can exist and, potentially, be fused, several challenges need to be addressed [20]. For instance, the semantics for the input modalities must be uniform: saying “left” or pressing the left arrow should result in the same semantic content, e.g., LEFT.

The adaptive, context-aware use of output modalities—fission—while it has deserved far less attention than fusion, can play a very important role in how interactive systems can adapt to different devices, users and dynamic contexts [21]. In this regard, very interesting contributions have been made by [22,23], dividing the life cycle of a multimodal presentation in stages, as also proposed by the W3C (generation, styling, and rendering [13,20,24]), proposing a mathematical model to determine the best possible combination of input and output modalities based on the user’s profile and preferences; and our group proposing the basis for an intelligent adaptation of output relying on context and user models—AdaptO [6].

### 2.2. Multidevice

Technology is widespread and the environments where we live and work are, unquestionably, populated by numerous devices, both stationary and portable [25]. It is not just a matter of the devices that exist, at certain places, such as wall displays, smart TVs, desktop computers, smart appliances or sensors, but also the devices users carry with them. This results in environments that are not only complex due to the amount of devices they contain, but also due to the degree of unpredictability of which devices will be present, at a certain time, their diverse characteristics, and the purposes they will serve. Therefore, Smart Environments need to go beyond multimodal interaction.

Designing for the multidevice ecosystem is a challenging task [25,26], particularly relevant due to the rise of mobile touch-enabled technologies [27]. In the past decade, several authors have addressed these scenarios to different extents. A few authors have worked on approaches for specific domains or applications, such as Visualization [28,29], the adaptation of media content in multidevice scenarios [30], tackling second screening scenarios [27] or interacting with different devices using a smarwatch [31].

To support the development of multi-device systems, different works have also been presented such as: HIPerFace [32] addressing multiple device integration; the web-oriented PolyChome [33] and Tandem Browsing Toolkit [34]; Conductor [35] and VisPorter [36], dealing with multi-display scenarios; adaptation of the user interface and interaction modalities depending on context [37]; Improv [38] and [39], supporting cross-device interaction design; PHASER [40], with a particular emphasis on a distributed architecture to support multi-device interaction; and the work described in [41], addressing end-user development of cross-device user interfaces. In this regard, our team has proposed extensions to the W3C MMI architecture to transparently enable multi-device support for applications by managing it at architectural level [19,42].

A few works also address the proposal of recommendations to design and develop multimodal and multi-device applications. As representative examples, Seyed [43] present a set of guidelines to improve user experience while interacting with multiple displays and Paternò [44] discuss relevant characteristics to be considered for the design of multi-device interfaces.

Despite all the advances made, in this context, several challenges have yet to be addressed to turn multi-device interaction from something happening in specific, predictable scenarios into something part of our everyday life, wherever we go and whichever devices are involved. Steps still need to be taken to provide a natural—i.e., intuitive and without long learning curves – multi-device user experience. Houben et al. [25] analyze the current panorama of cross-device interactions in the wild and identify a set of barriers still in place. In fact, devices and their applications are still rather oriented for personal single use and there is not much done regarding how devices know their ecosystem, i.e., which devices are there that can potentially be of use for the current context. On top of this, the guiding principles for managing content representation and action possibilities/capabilites across the expected heterogeneous range of devices are still scarce.

Dong et al. [26] also discuss the main challenges of designing and developing multi-device interaction and identify three main aspects that need to be addressed: (a) the proposal of methods for designing the interaction between devices; (b) the complexity of articulating interface design among different platforms, to cope, for instance, with platform-specific user interface standards; and (c) the evaluation of user experience in the complex setting created by multi-device interaction.

### 2.3. Sensors and Actuators

Sensors and actuators are a key aspect while creating a smart environment: sensors can capture and convert some aspect of the physical world to a digital signal, and actuators can convert a digital signal into some mechanical action [45].

Different types of sensors are available, capable of capturing a variety of aspects of the environment, such as motion, air quality, amount of light, temperature, humidity, door state, orientation, and location [45]. Some examples of actuators are smart lights, door locks, and smart valves to control water or gas flow.

There is a wide range of these devices and most need to be installed in some strategic location, which can result in communication problems. Most devices deployed in the context of smart environments use wireless sensor networks, with the adopted technologies and protocols depending on aspects such as range, bandwidth, and energy consumption. Most commercial devices use BLE (Bluetooth low energy), WiFi, and Zigbee protocols [46], the latter enabling the creation of a communication mesh taking advantage of multiple devices, thus facilitating the installation of some devices in farther locations, although the technology supports a low bandwidth [47]. These sensors and actuators communicate with other devices using a data structure according to the data they need to transmit ranging from boolean values (e.g., representing an open or closed door), to numerical values of temperature, or more complex data, e.g., for location.

### 2.4. Main Challenges

Stemming from our analysis of the relevant literature, notable aspects of which are summarized in the previous sections, and considering current research trends, there are a few key challenges that we deem relevant to drive research on how to improve interaction in smart environments:

- How to enable and support simple development of truly MMI applications, considering, for instance, the amount of technologies and complexity involved in deploying interaction modalities;
- In line with the previous point, how to simplify the exploration of fusion and integrate fusion engines in MMI frameworks, enabling full exploration of the multiple interaction modalities available;
- How to handle not only the devices deployed in the smart environment, but also those devices carried by users, which are natural candidates for interacting with the environment, and can become a common aspect when traveling between environments;
- How to provide a custom, user-adapted experience of the smart environment, addressing preferences and needs and, particularly, those aspects that might have a strong impact on the naturalness of the interaction, such as the user’s language;
- How to make multi-device interaction part of our everyday life, wherever we go and whichever devices are involved, in the sense that it should become an inherent feature of any application, and its implementation should be transparent to the developer.
- How to handle, in a unified, simple and coherent way, the different types and communication technologies of sensors and actuators for the smart environments, to enable a seamless interaction with the environment as a whole, and not a set of interactive isolated artifacts.

## 3. The AM4I Architecture

Considering the challenges previously identified, this section starts by presenting the set of requirements that guided our proposal of an architecture deemed suitable to address them. Without loss of generality, and to provide a more tangible shape to the Smart Environments scenarios we wish to support, we target that the architecture should be particularly suited for adoption in buildings with multiple spaces serving very different purposes, and a wide variety of occupants, such as a Research & Development Institute, and homes.

### 3.1. Main Requirements

The identified challenges lead to a set of requirements that should be considered for the design of an architecture for Smart Environments, and contribute to motivate adoption of the architecture by developers, entailing:

(1) modular design, and fully decoupled components.
(2) future extensibility, such as the creation of new modalities and components by adopting, whenever possible, standard formats, languages and approaches.
(3) easy to use and deploy, for multiple platforms, provide components and modules off-the-shelf, and be scalable.
(4) support multi-device interaction, both with devices installed in the smart environment, but also personal devices, such as smartphones, with a more transient existence, in the ecosystem;
(5) provide different ways of interaction and consider approaches to provide developers with high-level methods as an entry point to define and explore fusion of modalities (redundancy and complementary);
(6) user and context awareness to provide applications with the information relevant to adapt their features and those of the environment to particular user needs, preferences and mood;
(7) assuming speech interaction as a key aspect to contribute to a more natural interaction with the environment, and recognizing that, nowadays, multilanguage environments are quite common, support multilingual interfaces, both in visual and speech modalities.

### 3.2. Overview of Architecture

The definition of the architecture, considering the aforementioned requirements, profits from our previous work in designing an architecture to address Ambient Assisted Living scenarios (AAL) with a single end-user application for desktop and mobile devices [15]. For that context, our proposal was aligned with the Multimodal Interaction Architecture proposed by the W3C [13], but going beyond the target of web contexts specified in the standard.

To be usable in the targeted smart environment scenario the architecture needed to be extended to support multiple applications running in multiple devices capable of profiting from a dynamic set of input and output modalities and adapting to context and user.

**Figure 1:**
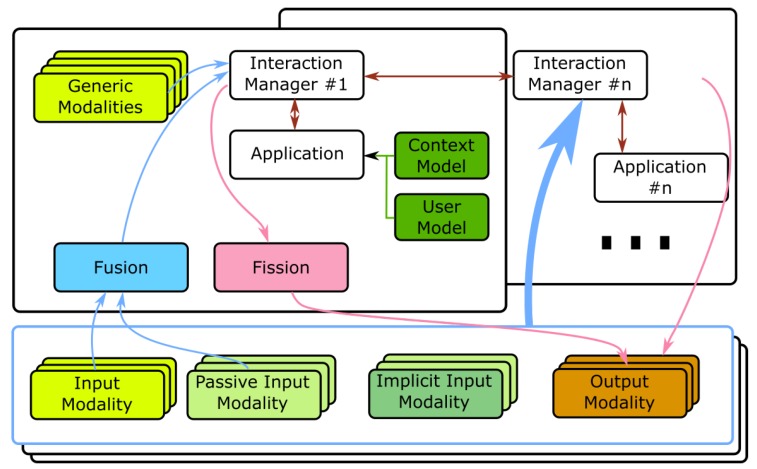
Proposed architecture: the core modules (e.g., interaction managers and applications) manage multiple modalities spread across different rooms/devices.

Figure 1 presents a diagram of the architecture depicting its main components covering several spaces distributed over two buildings, the main highlights being:

- **Modalities** are the components that support the communication (interaction) to and from the building. To comply with the modularity requirement, modalities are separate, decoupled processes. There are several types of modalities:
  1. Input: responsible for listening to the user’s commands (e.g., touch, speech, gaze) and generating events, coded with the MMI life cycle event markup as described in the W3C standard [13];
  2. Output: providing ways for the system to communicate with the user, transmitting any desired information and feedback (e.g., graphics, speech messages, text, sound);
  3. Mixed: gathering, in the same module, both input and output capabilities;
  4. Passive or Implicit: providing relevant interaction information to the system without an explicit action from the user and very important sources of information to feed context and user models (e.g., temperature, user’s location, affective state, comfort levels). Implicit modalities provide information regarding the user while passive provide information from the environment;
  5. Generic: enabling a complex interaction option, of-the-shelf, highly configurable, encapsulating a set of diverse technologies, and reusable in any application adopting the architecture (e.g., multilingual speech interaction, multimodal affective context)
  The modalities can run locally (e.g., on the occupant’s laptop) or in a hybrid setup, relying on support Services to perform more complex processing or enable their easier update (e.g., automatic speech recognition and natural language understanding [48], emotion identification). Legacy technologies can be integrated into the architecture, as modalities, by creating portals, as proposed in [49].
  An (input) modality can give feedback to the user without intervention of the application (and Interaction Manager) if it has the capabilities to do it. Since the architecture adopts the definition of modalities as proposed by the W3C, a speech modality, for instance, integrating input and output can directly provide feedback to the user by synthetic speech. If the modality does not integrate the needed output capabilities, it needs to generate an event requesting that the application delivers the feedback to the user through other means.
  The modalities should abide to a common semantics of events (e.g., say “left” or a “swipe left” gesture can have the common outcome of LEFT), adopting the ideas of Dialog Acts [50,51]. This way, as long as the semantics is kept, an application can seamlessly profit from modalities developed and/or integrated after its release, a very important feature for the building scenario with a longer timespan than technology.
- **Interaction Managers** are core modules in the architecture since they are responsible for receiving the events generated by the input modalities and producing new messages to be delivered to the output modalities. They can run locally (e.g., in the device held by the user), in the building servers or in the cloud. Multiple Interaction Managers can be used, by an application, and different applications can share the same Interaction Manager and, if needed, share modalities. It is also through the Interaction Manager that a single application can be transparently controlled by multiple devices, at the same time.
- **Fusion** is an important feature, since it allows multiple interaction events, coming from different modalities, to be combined into new single events aggregating all the information (e.g, point to a lamp—gesture identifying the target—and say “turn this on”—voice identifying the action). Fusion, in the AM4I architecture, is separated from the Interaction Manager, to allow the exchange of the module by other fusion modules and is actually where all modalities send their events for an early processing before the fused event is sent to the Interaction Manager. A common event semantics, among modalities is also important to simplify the specification of fusion rules.
- **Fission** consists in selecting one or more output modalities to deliver a message or content to the user [52] and is an important module for an increased adaptation to the occupant. Typically, in a multimodal environment, a set of output modalities is available and the system can choose from one or more modalities to render the message. The selection can be based on the type of message (e.g., text, numbers, warnings), availability of modalities (e.g., graphical display, speech output) or user context (e.g., alone, in the building lobby, in a meeting).
- **Context and User models** are essential for improving the level of adaptation provided by the system and can be available at building servers and/or the Cloud. They are available for use by multiple applications and this does not mean that only one monolithic general model is available. If needed, an application can have its own models, possibly obtaining part of the information from those pertaining a wider scope, and populating its own with more specific information. They must adopt standard representations to enable access by the applications without compromising privacy and security issues. The existence of user and context models does not mean that the occupants need (or will be forced) to disclose any of that information (e.g., location, affective state, facial features) to interact with the building and experience some base level of adaptation. Context and User models are there to complement and improve adaptation.
- **Communication, Standards, and Interoperability**, considering the required decoupling among architectural components, should rely on widely disseminated practices. The definition of the event transport layer, the communication protocol and markup languages for the communication between the Interaction Manager and the modalities inherit from the decoupled nature of the W3C multimodal interaction architecture specification and our proposal adopts markup languages (e.g., the Extensible MultiModal Annotation markup language—EMMA) used to transport notifications between components [13,53,54].
- The **applications** receive and process the events from input modalities; implement all the functional requirements; use and update context and user models to adapt; control services (e.g., central heating system) and actuators; and generate the output information to be conveyed to users using output modalities. They are executable programs that handle mainly the application logic, context and user awareness. User only interacts with the modalities, implemented as separate processes. The **Applications** can be single-device, one application running in multiple devices [19,55], server/cloud-based applications supporting features for (parts of) the building, or complex services consisting of different collaborative applications running at different locations.

**Figure 2:**
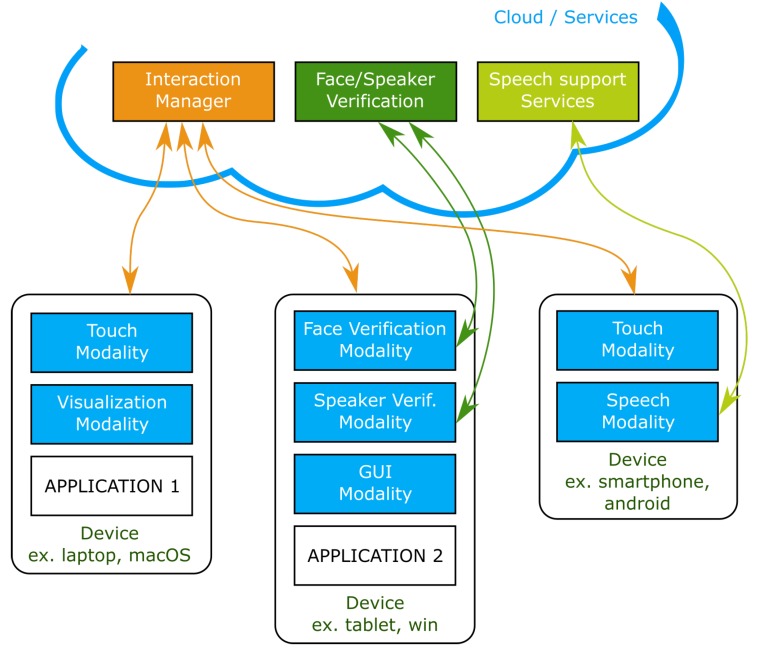
General view illustrating how modalities, applications and support services (in the building’s servers or the cloud) work together in an **Smart Environments** context. Different devices, applications and platforms are supported.

Figure 2 presents a general view of how modalities, applications and support services (in servers or the cloud) work together, in an Smart Environments context. Some devices may be running applications and include interaction modalities, but others can simply be the technological support for modalities. For instance, a smartphone (on the right of the diagram) might just be supporting the audio recording used to perform speech recognition to interact with Application 1, running on the laptop.

The described architecture includes modules to support **A**daptive, **M**ultilingual, **M**ultiplatform, **M**ultidevice, **M**ultimodal **I**nteraction, (**AM4I**, “Amphorae”, for short), as follows:

- **Multimodal Interaction**—The architecture supports a variable and dynamic number of input and output modalities by adopting an Interaction Manager assisted by Fusion and Fission services. These modules, together, can be seen as a macro Interaction Manager capable of receiving events from input (and passive) modalities, combining them (considering, for instance, a CARE model [5,56]) and sending information to output modalities.
  In the first approach, each device runs an instance of the Interaction Manager while, in the second approach, a single cloud based Interaction Manager runs remotely and each device connects to it.
- **Multidevice**—The proposal encompasses Interaction Managers, which are continuously running at building servers or the cloud, and all the interaction modalities know their location and communicate with them [15,17]. Complex devices, such as smartphones, integrate in the architecture manifold: as platforms to run applications, as (several) passive modalities, and providing multiple input and output modalities.
- **Multiplatform**—One important characteristic of the architecture, originating from its decoupled, distributed nature, is the ability to encompass multiple platforms. The Interaction Managers, Fusion and Fission modules are generic and available as services allowing applications to run in any platform, such as iOS, Windows, Android, or Linux variants, as long as they abide to the communication protocols.
  It is also possible to develop modules that run, without changes, in different platforms, and browsers are strong candidates to support these applications, since they run in almost every platform by, for instance, adopting an approach based on HTML5 (https://www.w3.org/TR/html5/)/Javascript applications.
- **Multilingual**—As a first approach, generic modalities are the architecture components contributing to support interaction adapted to users culture. An important example is the included generic speech input modality supporting spoken commands in different languages, taking the user profile into account, as proposed in [17,57].
  Other parts of the architecture contributing to multiculturalism are the user models and passive modalities providing information on cultural aspects, such as, for example, the languages spoken and understood by an occupant. A passive modality considering, for example, information on the most commonly used dictionary in a smartphone, might help selecting the language for speech input and/or output.
- **Adaptive**—To support adaptation, the architecture provides:
  (1) models and services for users and contexts, that can be considered to adapt the interaction with the applications. (2) integration and gathering of contextual information, including information from different sensing technologies and building installed systems (e.g., security cameras can support the determination of user location); (3) output adaptation considering user and context. Passing information to the user is very important and must make the best possible use of the available modalities taking into consideration context and user. The architecture handles this by adding adaptability to output modalities and a dynamic registry of available output modalities taking into account the context and user, as proposed for AdaptO [6].

## 4. The AM4I Framework

To be of use, the architecture presented in the previous section needs to be instantiated. Extending previous work for multimodal interaction in AAL scenarios [15,16] the AM4I framework has been developed, enabling concrete deployment of multimodal interactive capabilities in Smart Environments.

**Figure 3:**
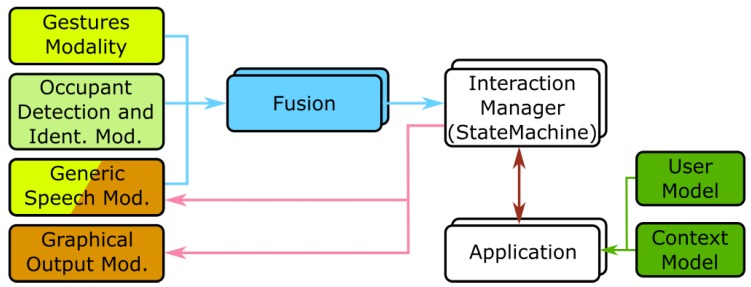
Diagram depicting the modules included in a first instantiation of the AM4I architecture for Smart Environments: the AM4I Framework. Multiple instances of each module can coexist.

The framework, depicted in Figure 3, closely follows the proposed architecture and this first version, albeit not fully implementing all the modules proposed in the architecture, already provides a wide range of features enabling the instantiation of interaction designs. At this stage of development of AM4I, fission is limited to basic functions, such as distribution of content to the available output modalities. These functions are implemented by the Interaction Manager and the application.

To achieve the goals for the proposed framework (AM4I), each module is essential: the context and user model, along with a wide range of off-the-shelf interaction modalities support **adaptation** of the interaction; modalities are decoupled, developed using methods to enable running them in **multiplatform**; the generic modalities proposed offer embedded **multilingual** support; the Interaction Managers, the decoupled modalities, and the distributed nature of the different components support **multimodal** and **multi-device** systems.

Overall, modules communicate using HTTP and W3C MMI life cycle events [13]. These events provide a standard communication language for the Interaction Manager to invoke the modalities and receive events.

In what follows, we provide a description of the main components, providing information on the adopted technical solutions and capabilities of the existing modules.

### 4.1. Modalities

As previously argued, we consider that the adoption of the architecture and framework depends on the existence of a set of off-the-shelf interaction modalities that enable developers to deploy applications without the need to master the involved technologies.

In its current development status, the AM4I framework includes, off-the-shelf, several generic modalities (e.g., speech, gestures), some passive modalities and a small set of other modalities. More modalities are in development, including: a decoupled touch modality integrating detection of touch in a device different from the one used for rendering the graphical content of the interface; a gaze modality; and a silent speech modality. The following subsections describe representative examples of each kind.

#### 4.1.1. Generic Speech Modality

Due to its relevance for hands-free and interaction at a distance [58] a generic speech modality is part of our framework since its genesis, serving as a first example of what should be a generic modality.

The language dependency of speech related modalities is one of the main challenges to develop a generic speech modality. In the case of speech recognition, models and grammars must exist for each desired language. Our overall purpose was to provide a generic speech modality that supported, as best as possible, all the resources, configurations and specific customizations to enable easier development of multilingual applications. This modality features speech input, i.e., automatic speech recognition (ASR) and speech output, i.e., speech synthesis.

#### Speech Input

**Figure 4:**
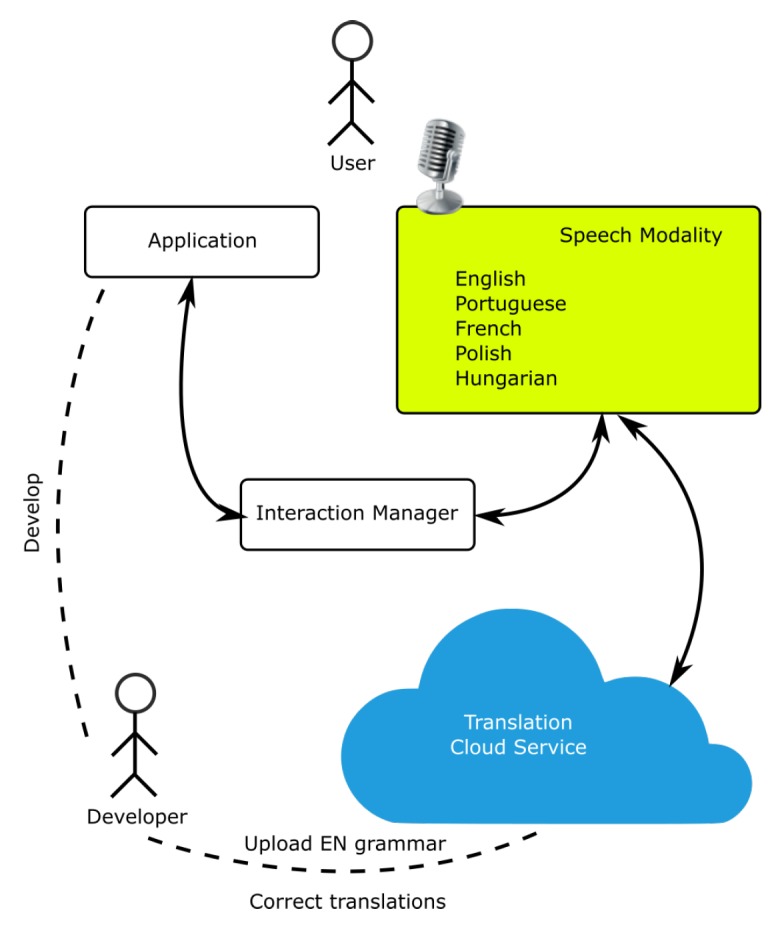
Overall illustration of multilingual speech interaction design. The developer uploads a grammar in English and a translation service generates grammars for other languages.

The speech modality supports two different recognition modes: the recognition of commands and dictation. The first is supported by a speech grammar and the second by a dictation model using a statistical language model. The modality is capable of switching from recognition of commands to dictation in runtime, whenever it receives a request from the Interaction Manager. When the modality is loaded and sends the NewContextRequest life cycle event to the interaction manager it receives the context of the application and information regarding the current language. Then, it must be configured with the corresponding grammar for that language. The proposed solution to tackle multilingual support was the creation of a cloud-based translation service [59,60]. Figure 4 shows the architecture and communication between the modules. The service is capable of automatically translating an English grammar, uploaded by the developer while developing the application, into the target languages. To translate the grammar, all possible sentences that can be extracted from the English grammar are extended. Then, using Microsoft Translator Text API (https://www.microsoft.com/en-us/translator/translatorapi.aspx) all sentences are translated and the grammar is reassembled maintaining all the original rule names. Since translation is not perfect, or if the developers want to add other versions for the translations, a web site is provided to enable verification and manual changes. The service also supports the inclusion of dynamic rules so that the grammars can be changed in runtime. This enables the recognition of specific dynamic content of the application.

Whenever the user interacts with the modality and speaks any sentence that is recognized by the speech engine, the modality requests the translation service to extract the meaning of the sentence, i.e., its semantic content. The modality includes Spoken Language Understanding (SLU) and it uses the Phoenix parser [61] that, given a recognized sentence and the grammar, can extract the semantic information of that sentence.

The semantic information is defined by a semantic layer based on dialog acts and the produced semantic output will be the same regardless of the input language.

Speech recognition (at client or server side) uses Microsoft Speech Platform, that offers recognition for several languages.

#### Speech Output

The languages supported for speech output are the same as for speech input. When the multimodal system needs to transmit something to the user, through speech, it sends a message to the modality encoded in Speech Synthesis Markup Language (SSML). This markup language supports the encoding of the content and other speech related parameters. For speech synthesis a speech engine is used that already supports voices for several languages, but most of the languages are limited to one voice. Therefore, to expand the set of choices for European Portuguese, additional voices were recorded and trained [62], providing users the possibility to select a particular voice (choosing the gender and young or older adult), according to their preferences.

#### 4.1.2. Modality for Occupants Detection and Identification

This modality uses a Microsoft Kinect One to track the occupants of an office (or equivalent space) and detect changes in their number—detecting arrivals and departures—and provides IDs for the occupants based in face identification.

The modality, after being initiated by a message from the Interaction Manager, continuously tracks persons and the changes in their number. When the number of detected persons increases, the modality tracks the face and identifies the person using (although this can be replaced by any other equivalent or custom service) the face API from Azure cognitive services [63].

The information from this modality (and other passive or implicit modalities) is sent via Interaction Manager to a context and user management application in the building servers, which will feed the context model with information (number of persons in a certain place and their IDs). This information will only be available to applications and other users according to privacy policies, including the need for user authorization.

This is an example of a modality that feeds the system with implicit interaction and other examples regarding room temperature or other comfort related measures might easily be instantiated.

#### 4.1.3. Graphical Output Modality

The Graphical Output modality is responsible for dealing with content display mainly rendering text and simple graphical information. One important aspect to emphasize, regarding graphical output, it that it does not need to be tied or limited to being supported by the display of the device where the application is running. Given the decoupled nature of the architecture, an application can be running on the occupant’s smartphone and the graphical output modality (e.g., to show a graphical user interface) can, for instance, be supported on a laptop’s display, or a projector.

The graphical output modality receives text, images and associated type of presentation to be considered (e.g., dialog box) and uses simple templates to render it.

#### 4.1.4. Gestures Modality

A first implementation of a generic gestures modality is also available, capable of recognizing users’ body gestures. Using Kinect technology, it is possible to track a person’s posture, recognize the hand, and follow the movement of the bone joints. The recognized gestures include, e.g., hand swipes (right, left, up, and down), and their use is particularly interesting if the user wants to scroll some content displayed in a screen and is away from other input device or is not in range for speech commands.

### 4.2. Interaction Managers

The Interaction Managers are modules developed in Java that implement an HTTP server to receive events from input modalities. The events are parsed and processed using a state chart machine, defined using SCXML, adhering to the W3C recommendation [64]. In this regard, we considered the Commons SCXML library, from Apache Commons [65], and extended it to implement the desired instructions for some elements of the SCXML such as the send element.

**Figure 5:**
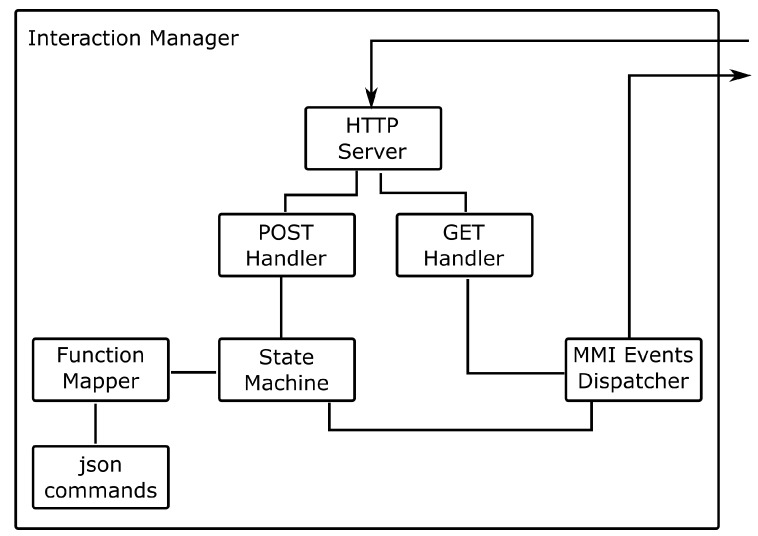
Main internal components of the Interaction Manager.

Figure 5 presents the internal components of the Interaction Manager. The main instance of the Interaction Manager initiates the SCXML state machine and an HTTP server containing one handler for POST messages and another for GET messages. When an event is received by the server, the POST handler parses the event and triggers the state machine. The Function Mapper contains the methods that can be invoked from the SCXML state machine. Sending events generated in the state machine to modalities is the responsibility of the MMI Events Dispatcher, which sends a request with the life cycle event, if the modality has an HTTP server, or responds to the polling of the modalities. When modalities poll the Interaction Manager, the GET handler waits for a timeout to respond with a renew message. If an event needs to be sent to that modality the GET handler passes the response stream to the MMI Events Dispatcher.

The basic SCXML used to configure the Interaction Manager includes two parallel main states, one to receive newContextRequest from modalities and set the modality to available state, in that context, and other to receive the notification of events resulting from user interaction.

A single instance of an Interaction Manager can be responsible for managing several modalities and support multiple client applications. Each exchanged message is associated with a client identification so that each running instance of the Interaction Manger is capable of running multiple state machines, one for each client.

All modalities and applications expect to communicate with an Interaction Manager. In the current development state of AM4I, the Interaction Managers and Fusion modules have known addresses and ports, which are passed to the modalities and applications, at their start. Modality registration is handled by the Interaction Manager and developers can manually or programmatically define the modalities with which an Interaction Manager communicates.

### 4.3. Fusion

**Figure 6:**
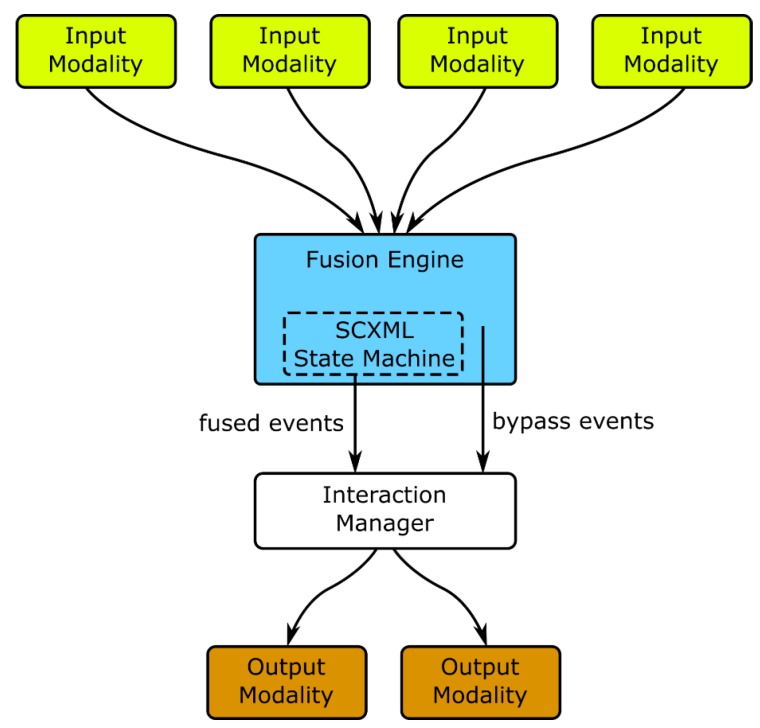
Multimodal Fusion Architecture. Input modalities send events to the fusion engine, the events can be fused into new ones or the fusion engine can directly pass them to the Interaction Manager. Then, the Interaction Manager interprets the events from modalities and the fused events.

Fusion is strongly related with the Interaction Manager in the sense that they constitute the two corner stones of dealing with interaction inputs. Input events are actually sent first to the fusion module and only the outcomes of event fusion (or single events if they are not configured to be fused) reach the Interaction Manager. In our architecture, Fusion is, conceptually, a part of the Interaction Manager, but decoupled from its core, as presented in Figure 6, to allow the exchange of the module by other fusion engines.

The main objective of our fusion module is to simplify the process of creating and including fusion of events in new applications, removing some of the complexity when designing multimodal interaction. Basically, the module works similarly to the Interaction Ianager and the core is still an SCXML state machine, which receives events and sends events to the main Interaction Manager. A particular aspect that should be highlighted, in our proposal, is the method to configure the fusion engine supporting the creation of the corresponding SCXML. In a system with many events and fusion points, the creation of that SCXML is complex and we considered that some method should be provided to enable its definition at a higher (more conceptual) level.

**Figure 7:**
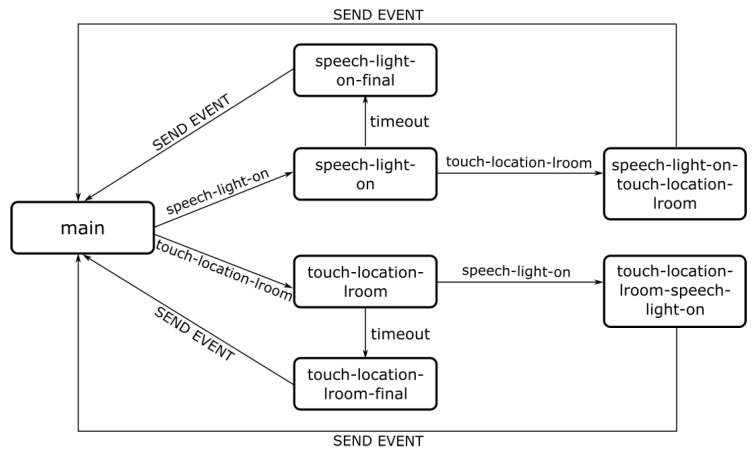
Example of a simple state machine for fusion of two events.

In our method [66], all modalities publish all events that they can generate by creating a file with a predefined structure. Also, a file with the same structure must be created to define the output events. The generation of those files is done according to the syntax of a programming language, enumerating all the relevant information. Additionally, a class is proposed supporting the different operations required for fusion, enabling the definition, by the developer, of which events to fuse and how. By importing the files generated for each modality to an integrated development environment (IDE), all the features of the IDE are available, such as autocomplete or syntax suggestions. These features help the developer to create a set of lines of code that are a high-level description of the intended combination of events. Compiling that code automatically generates the SCXML file to configure the fusion engine. Figure 7 illustrates a simple state machine for fusion of the events (speech-light-on and touch-location-lroom) coming from speech and touch modalities.

In order to have coherent events, we define a semantic layer based on Dialog Acts [50,51], extending the use to every modality, even for non-speech related modalities, resulting in the standardization of the semantic output. For instance, a swipe left touch event and a speech event of “turn left” can produce the same semantic output (e.g., LEFT). This approach reduces the complexity of defining the fusion rules.

### 4.4. User and Context Services

Only a simple implementation of User and Context modeling has been integrated in the framework, so far, mainly to address the needs of concrete applications such as the proof-of-concept scenarios presented ahead, in this article.

**Figure 8:**
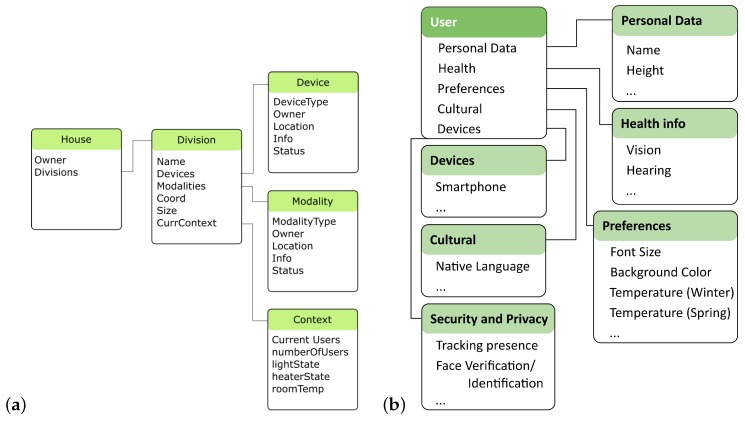
The framework Integrates, at this stage, (**a**) simple context and (**b**) user models.

The user information is available through a cloud service that, when requested, queries a database for the user and his model. The database is flexible and stores JSON-like documents, allowing to add new parameters in the future. Our first user model includes some information of the users (e.g., age and language) and preferences (e.g., preferred room temperature), as depicted in Figure 8b). The Context Model, also available as a service, illustrated in Figure 8a), is implemented using the same database approach. For the time being it stores information from passive modalities such as occupancy of building rooms, temperatures, lights and an history of application usage.

## 5. Deploying AM4I in Smart Environments: First Results

The AM4I Framework is being advanced, applied and subject of first tests in two real contexts of Human Building Interaction (HBI): (1) a non-residential building and (2) a smart home. In this section, we briefly present the two contexts, and representative examples of proof-of-concept applications for both. The system deployed in the non-residential building is at an earlier stage of development than in the smart home context.

### 5.1. Non-Residential Smart Building

Adopting our University Campus as our testbed and, particularly, the main building of the authors’ research institute (3 floors with large common-spaces, labs, and offices), we are exploring, in the scope of Project SOCA (Smart Open Campus), how technology can enable a smart interactive environment for the different occupants of the campus. One of the concerns, in this context, is how building lobbies, meeting rooms, offices and classrooms, for instance, will integrate interactive technology that will provide access to the different controls and services. Therefore, we are using the proposed architecture and framework to provide support to instantiate interaction designs enabling enhanced interaction between end users and buildings.

A shared space, a Laboratory, was adopted for the deployment of the developed solution and equipped with the the required hardware. A small computer, with a Microsoft Kinect One attached, was used to deploy a passive modality responsible for the detection and identification of the users (described in Section 4.1.2) and a speech modality. These modalities use the Kinect One integrated sensors (microphones, RGB camera, and depth camera) for capturing the users’ speech, body posture and face. The setup also includes a server, located in the building data center, running the Interaction Manager and the main application, which communicate with the user model and context model. Additionally, users can also use their smartphone or tablet. In concrete, the smartphone runs a speech modality, a Graphical output modality and a touch modality. Support services are running in the cloud or in the building server.

For the first proof-of-concept, we selected (1) the adaptation of non-residential building communication output to the user and (2) intelligent control of environment. The first resulted in user-adapted content in large displays now ubiquitous in all modern buildings; the second resulted in handling of lights and temperature control in offices. Both are described next.

#### 5.1.1. Proof-of-Concept 1—User-Adapted Content

The objective is to provide content adapted to a visitor or a researcher of the institute. For that, the system detects a person, tries to identify her and, then, using a large display, provides content aligned with the interests of that person.

**Figure 9:**
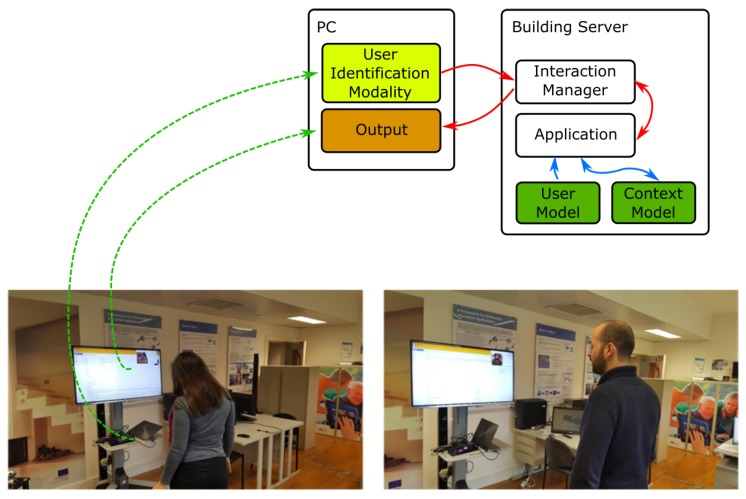
Photos illustrating the proof-of-concept 1, where content is shown to a lab occupant based on information regarding his/her interests. The diagram at top right depicts the main components of the A4MI Framework in use.

Figure 9 presents photos illustrating a real user testing this proof-of-concept, complemented by a diagram with the main framework modules involved.

In this example, there are two modalities involved: the user identification, as an input modality, and an output modality, which will present content according to the users’ interest based on information obtained from the user model.

When a user enters the lab, and is recognized by the identification modality, the modality sends an event to the Interaction Manager which forwards the event to the application. The application interprets the event and queries the user model for information regarding that user, creating an action to update the context that is displayed by the output modality. When a second user arrives, the system behaves similarly and the context of the modality is updated.

#### 5.1.2. Proof-of-Concept 2—Meeting

In the second example of use, a researcher receives someone in his office. Assuming that information about the visitor is already available from the building’s user model (whether specifically built, for the building, or populated from a more broad context, e.g., other interactions with other buildings), when the system identifies the visitor and verifies his/her preferences, it can act according to any predefined criteria or leave room for the office owner to decide For instance, if the preferred temperature for the visitor and owner is different, the system can ask the owner if he wants to adjust the room temperature through a notification presented in his/her smartphone.

**Figure 10:**
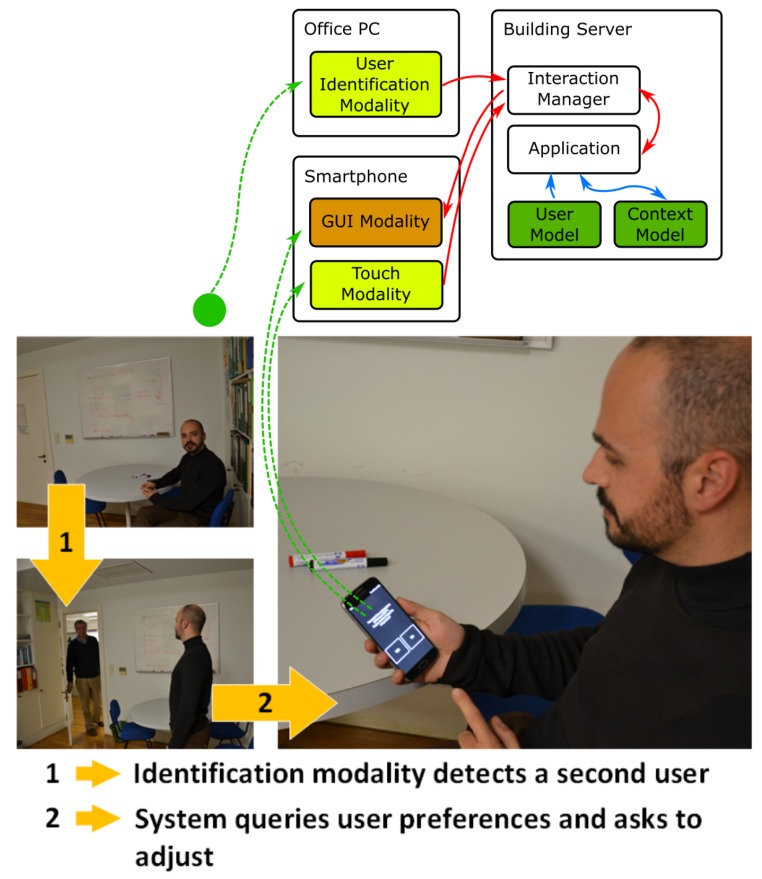
Illustrative scenes and framework components diagram for Proof-of-Concept 2—Meeting. A new occupant arrives at the office, for a meeting, and has different room temperature preferences than the first occupant (owner of office) who receives a notification to change temperature settings, if he wishes.

**Figure 11:**
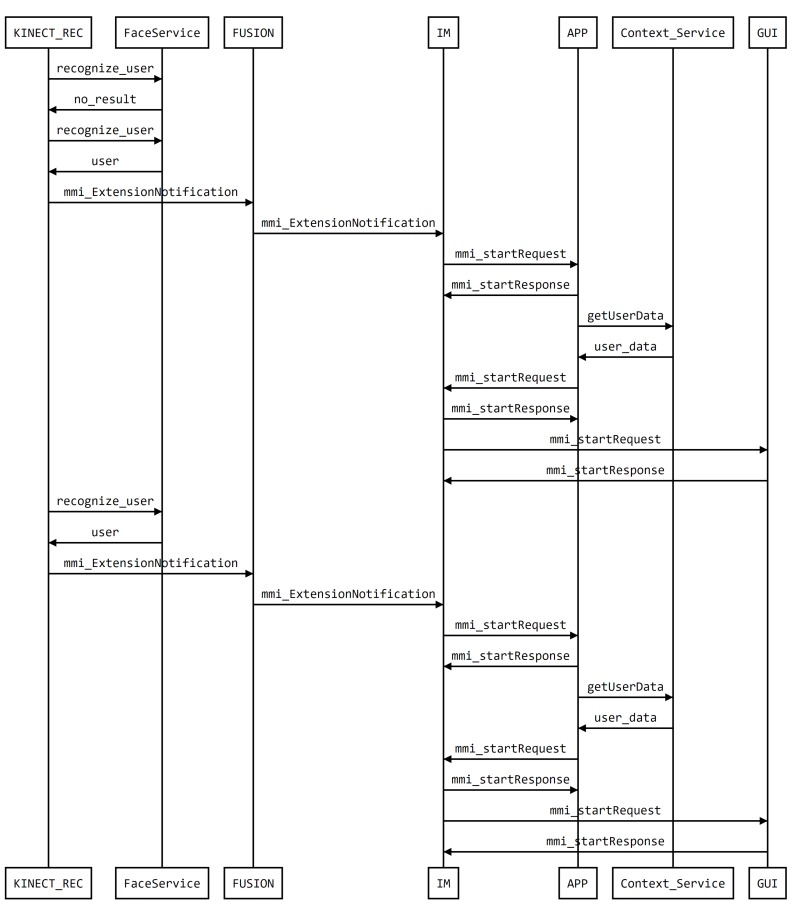
Sequence Diagram of the messages exchanged by the framework in the execution of this demo.

Some photos taken during a trial with real users are presented in Figure 10 along with a depiction of the intervening components. On the Framework side, Figure 11 presents the sequence diagram obtained from logging the execution of the demo. The diagram illustrates the modules of the system, modalities, fusion, Interaction Manager (IM), application, and other support services as well as messages exchanged between the modalities and the fusion and IM, implemented with MMI life cycle events.

In this proof-of-concept, the passive identification modality detects a new user and informs the application in charge of managing the context model, through the Interaction Manager. An Assistant, running on the building servers, in charge of managing users’ comfort, receives this information, from the Context Model (using a subscription for updates), and queries the users when it detects that the temperature preferences of occupants of an office are different, which creates a potential conflict. The Assistant initiates interaction with the office owner to decide the settings by sending him a notification, on his smartphone, with the question and a set of possible answers, e.g., settle on an intermediate temperature, which the user can select using touch. Naturally, this illustrates a simple preferences clash situation and based on a wider comfort context (i.e., beyond just temperature) the Assistant might propose additional (adequate) alternatives. Regarding the smartphone as the provider of the modalities considered for the notification, even though speech interaction might have been used, choosing the smartphone reflects the more “private” nature of this action that can, in some sense, configure a gesture of courtesy towards the other occupant.

### 5.2. Smart Home Context

The scenario adopted is a Smart Home, including: (1) command and control; and (2) better communication between the house and its users. Accordingly, our purpose is to deploy a home multimodal assistant ready to accept user commands and respond to information requests related to the house.

**Figure 12:**
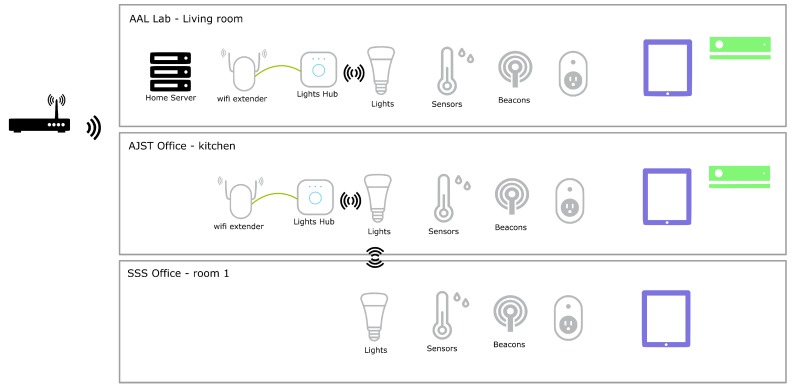
Smart home physical installation, showing the several sensors and devices deployed at each of the 3 spaces integrated the virtual home.

To deploy our prototype, we have created a small virtual house where each room has, at least, smart lights that the assistant can control. The virtual house made use of 3 different spaces in authors’ institute, simulating a living room, a kitchen and a room. The diagram, in Figure 12, presents the devices installed, in each office.

The living room, which usually is the room people spend most of their time, has the largest number of devices: it has sound and projector to deliver information to the user; a Kinect, as means to interact and for the home to recognize the user; and a heater controlled by a smart plug. A tablet can be used everywhere and can be moved between rooms. Additionally, several sensors provide data regarding temperature, humidity, luminosity and air quality.

Users can interact using several modalities and devices, such as a tablet, and interact using speech or touch. The home server is a central key for communication, interaction and smart home support services.

#### 5.2.1. Proof-of-Concept #1: Controlling the Lights in Any Division

The assistant can, for example, control the lights, either in the room where the user is or in any other. To turn on the lights, in another room, the user can consider different modalities made available in a tablet to, for instance, say “turn on the lights” and touch the house division in the blueprint displayed in the screen. In this concrete situation, the speech and touch events are fused together creating an event requesting the home assistant to turn the light in the living room.

**Figure 13:**
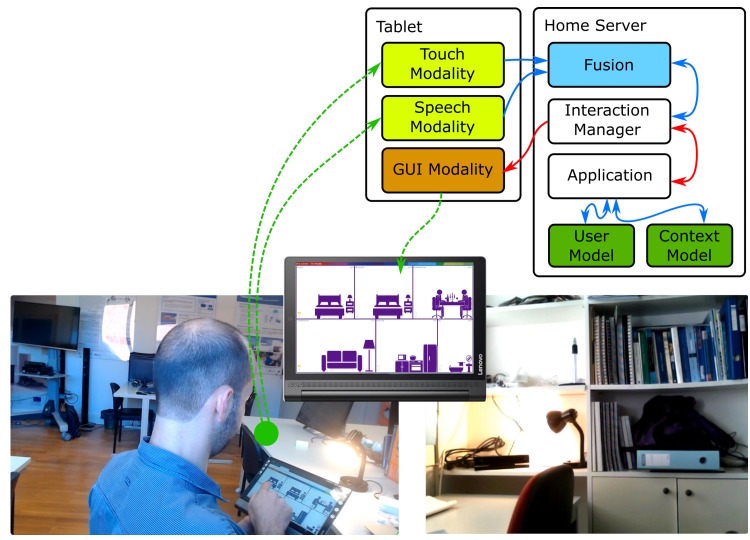
Images illustrating the use of the framework and assistant in the smart home context, while using fusion of speech and touch to turn the light on a different house division.

**Figure 14:**
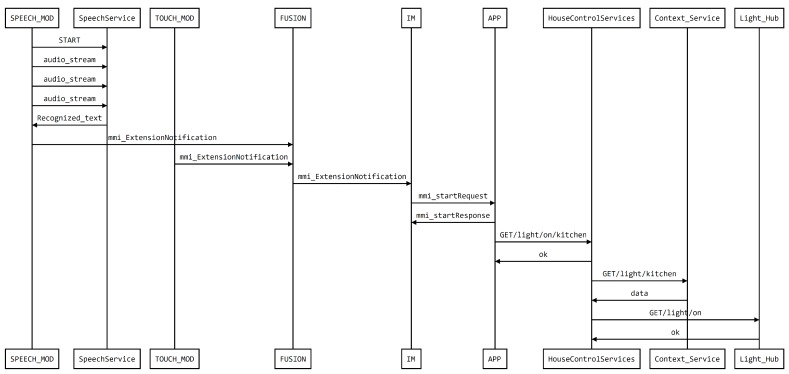
Sequence diagram of the communication between the different components of the framework, while turning on the lights, in the living room, when the user is located in another room.

Resulting from a real use situation with the described proof-of-concept, Figure 13 shows a representative set of images obtained during the execution of the described task, by one of the authors. The first image shows the user in the lab using the tablet; the second the effect of the command to turn on the lights in another room (simulating the kitchen); the third the tablet display, showing the status of the light. Illustrating the inner-workings of the deployed solution, Figure 14 presents the diagram of the messages exchanged among the different framework modules and application to execute the task.

This proof-of-concept exemplifies the use of a context-specific external service, HouseControlServices. Control and access to new features can be added by REST webservices, the adopted and recommended approach.

Also, at any time, the user can request the assistant for information regarding the state of the lights in a specific room or which lights are on by asking, for instance, “which lights are on?”. In this case, the assistant responds using speech synthesis.

#### 5.2.2. Proof-of-Concept #2: Accessing House Information

**Figure 15:**
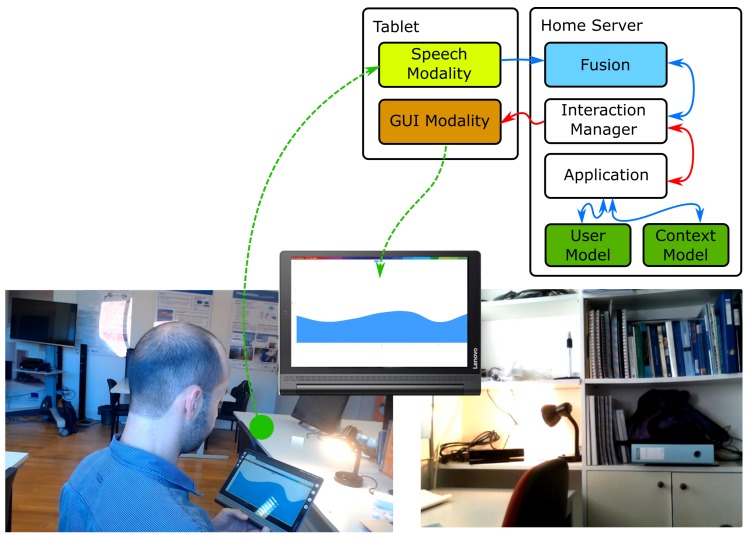
Images illustrating the use of the framework and assistant, in the smart home context, while using speech and GUI to obtain information regarding the consumption of water, in the house.

Another example of usage of the assistant is to access information regarding the consumption of resources, in the house, such as water or electricity. Figure 15 illustrates the execution of such task.

The user wants information on the water spent, in the previous week, and utters “what was the consumption of water last week?”. Them, the Assistant, using a graphical output modality, presents a graph to the user with the consumption during the week. The user can also ask about the consumption of electricity or gas, and in other periods of time, such as the day or month before.

## 6. Conclusions

In this article, we propose an architecture and its instantiation, the AM4I framework, contributing to an easier deployment of interaction designs. The current stage of the framework already addresses several of the key issues identified as paramount to advance smart environments from an Interaction Design perspective, and several illustrative proofs-of-concept, for simple scenarios, show how it can already provide support in realistic situations.

The current stage of the framework is the result of several iterations improving and adding new features. One important aspect of these iterations was that, for each, a concrete application was targeted, allowing a clear definition of requirements and the assessment of how well the framework served their implementation.

The proposed architecture and framework tackle, albeit to different extents, the challenges and requirements identified as leading goals for our research. The AM4I framework already supports the development of MMI applications by providing a set of modules (e.g., interaction manager) and off-the-shelf interaction modalities (e.g., multilingual speech) that developers can add to their applications. This means that, for instance, a developer without knowledge about speech interaction can add it to an application, or multiple applications can rely on the same exact implementation of an interaction modality. The decoupled nature of the different modules and modalities is ensured by the adoption of a communication based on MMI life cycle events and its versatility is confirmed by the growing number of off-the-shelf modalities being proposed without entailing any change to the existing framework modules. The multimodal nature of a system only shows its mettle when interaction modalities can be fused to serve more complex (and natural) forms of interaction. In this regard, AM4I encompasses a fusion module and, more importantly, proposes a high-level method for defining fusion patterns, i.e., how to fuse the different modalities available. Regarding the integration of all available devices, including personal devices, the framework, given its decoupled nature, enables their inclusion as additional modules bringing, e.g., novel modalities or new applications to the smart environment. Since the support for multi-device is embedded, at the architectural level, the same application, running on multiple devices or modalities dispersed over different equipment can be seamlessly used.

Regarding user and context awareness, AM4I already supports some basic features regarding the consideration of user and context information important, e.g., to react to user presence. Additionally, the framework already encompasses an affective modality, enabling that the ecosystem is aware of the user’s emotional status. Finally, recognizing language as an important resource for a more natural interaction with the smart environment, and the increasingly common occurrence of multilingual communities, a strong effort has been put into providing, off-the-shelf, the means to automatically support multiple languages, a capability generated dynamically from the configuration for one of the languages (e.g., English). This effort also illustrates the potential of generic modalities in providing developers an easier access to advanced technologies for interaction design.

The capabilities and versatility of the architecture and framework were demonstrated multiple times, notable examples being: (1) their use to create multimodal complex systems and modalities, e.g., a multimodal interactive speech-gaze personal assistant [67]; (2) their adoption as the architecture and framework for the development of a multimodal assistant in the scope of European project AAL PaeLIFE [68,69], involving multiple development teams spread over Europe; (3) their use, as the basis for a graduate course, by the authors, in Multimodal interaction, for several years (in their multiple iterations). As an example of the works developed by students, the reader is forwarded to [70], describing a multimodal presentation system made possible by the AM4I framework and developed in a few weeks of work (illustrative video available at https://youtu.be/pcbRcsfxFX4).

Overall, and considering the different features of the proposed architecture and framework, such as its decoupled nature or its proposal of generic off-the-shelf modalities, it has a strong potential to evolve, further addressing aspects such as improved adaptation to user and context.

Additionally, and in line with the definition of interaction design [71], it is important to note that attention to user needs and motivations must provide the requirements for what is designed and deployed. In Nabil et al’s words [72], “if studies of smart homes have told us anything, it is that we need to understand people and how they want to live their lives before we can really understand how technology can best be designed to suit them.” In this sense, user-centered design considering tools such as Personas, scenarios [71,73] and user stories [74,75], can be an important asset to support a multidisciplinary user-centered dialogue in smart environments. These, can provide a common language among the different research areas similarly to what we have proposed for multidisciplinary collaboration in designing for children with autism spectrum disorders [76,77]. And, in this context, the characteristics of the AM4I framework will, we argue, further show their mettle, supporting iterative design and development of systems, in short prototyping-evaluation cycles, which can further contribute to refining the requirements and increasing our multidisciplinary insight to advance smart environments. Note, for instance, that adding or removing support for a particular interaction modality is a task not entailing any change to the application core.

### Future Work

The versatility of the proposed architecture and framework, and their alignment with the key aspects needed to provide improved Interaction in smart environments scenarios, naturally open several routes for further work. Among them, we identify a few that both highlight the potential of our proposal and would, in our opinion, more significantly serve smart environments.

As argued, the number of modalities made available, off-the-shelf, is a pre-condition for the adoption of the framework, by developers. It is important that the deployment of a particular interaction design does not depend on the developers mastering the required interaction technologies. Therefore, evolving the set of generic modalities available in AM4I is of the utmost importance and we are actively working to expand the set of modalities provided by the framework. In this regard, we have a strong focus on speech interaction with research ongoing on silent speech interfaces [78,79] and articulatory-based photo-realistic audiovisual speech synthesis [80]. Additionally, the expansion of the framework with a more advanced generic affective modality would enable richer user contexts to foster further adaptability also entailing additional evolutions of the fusion engine. The initial results of this line of work were presented in [81].

To support additional modalities and services the mechanisms for modality registration and discovery need to evolve to profit from recent work, in the scope of W3C MMI architecture, such as [82]. The alignment of AM4I with the W3C recommendations facilitates profiting from these recent efforts.

Adaptation to user needs, expectations, preferences, and context (e.g., task, location, group) is an important aspect to consider in smart environments. The proposed architecture contemplates these aspects relying on contextual information from users and on fission, i.e., the ability of the system to make smart choices on how to better communicate content to the occupants. The approach instantiated in the AM4I framework is still very simple, both regarding the user and context models and adaptation, and the work needs to evolve to fully embrace the proposed view integrating, for instance, a model inspired in AdaptO [6] and contributions from the Social Sciences (e.g., mental models [83] and context and occupant behavior [84]). In this regard, occupant identification and location, useful in the context of adaptation, are, given the dynamic nature of smart environments, important and challenging features (as an example, see [85] for a recent review covering identification on multi-touch surfaces) that should, in our perspective, be treated with care, subject to the occupant’s explicit authorization, and not a major requirement for a natural adaptive interaction with the building, i.e., systems should not be designed around occupant identification, but benefit from it, if available. Users’ identification, contextual information and output adaptation should be explored to handle interaction privacy, avoiding, for example, use of speech output for confidential information when the user is not alone.

Regarding the evaluation of user experience in smart environments contexts, we propose the integration of an evaluation infrastructure with the multimodal interaction architecture. Our previous work on this matter has resulted in DynEAAS—Dynamic Evaluation as a Service [86,87]—supporting context-aware evaluations and fostering evaluation in dynamic environments. Nevertheless, to bring evaluation into a large number of systems, the evaluation services should easily be a part of every application developed based on the AM4I framework, which entails an additional effort to integrate DynEAAS into AM4I.

These and other related lines of research are being addressed in the scope of two ongoing projects providing the scenarios and requirements to advance the AM4I architecture and framework. In a more controlled context, project SGH – Smart Green Homes, a partnership with BOSCH, is addressing interaction design for the SmartHome ecosystem. In a broader scenario, entailing stronger diversity of spaces and requirements, project SOCA – Smart Open Campus – is contemplating how an University Campus can be harnessed with technologies and services that improve its occupants daily routine, the challenge being, for our team, on how to design and deploy the interactive natural access to such features.

## Abbreviations

The following abbreviations are used in this manuscript:

AAL	Ambient Assisted Living
AM4I	Adaptive Multiplatform Multidevice Multilingual Multimodal Interaction
API	Application Programming Interfaces
ASR	Automatic Speech Recognition
BLE	Bluetooth Low Energy
CARE	Complementarity, Assignment, Redundancy and Equivalence
EMMA	Extensible MultiModal Annotation markup language
HBI	Human Building Interaction
HCI	Human Computer Interaction
HTML5	HyperText Markup Language5
HTTP	Hypertext Transfer Protocol
IDE	Integrated Development Environment
IM	Interaction Manager
JSON	JavaScript Object Notation
MMI	MultiModal Interaction
SCXML	State Chart XML
SGH	Smart Green Homes
SLU	Spoken Language Understanding
SOCA	Smart Open Campus
SSML	Speech Synthesis Markup Language
W3C	World Wide Web Consortium
